# 3D holographic reconstruction and intraoperative navigation combined with CBL teaching in laparoscopic partial nephrectomy training for urology residents

**DOI:** 10.1007/s13304-025-02466-w

**Published:** 2025-12-11

**Authors:** Cunyao Li, Xiaoliang Yang, Wei Qi, Yu Wu, Xu Yan, Yizhe Wang, junfeng Jing, Can Wei, Yanbin Zhang

**Affiliations:** 1 Department of Urology, The Second People’s Hospital of Hefei, Hefei Hospital Affiliated to Anhui Medical University, Hefei, Anhui China; 2 The Fifth Clinical School of Medicine, Anhui Medical University, Hefei, Anhui China

**Keywords:** Renal tumors, 3D digital holography, Case-based learning (CBL), Standardized residency training

## Abstract

**Objective:**

To evaluate the effectiveness of integrating 3D digital holographic reconstruction and intraoperative navigation with case-based learning (CBL) in enhancing the educational outcomes of laparoscopic partial nephrectomy training for urology residents.

**Methods:**

A prospective randomized controlled trial was conducted at the Second People’s Hospital of Hefei from August 2021 to May 2024. A total of 120 urology residents were randomly assigned to either a combined teaching group (3D holography + CBL, *n* = 60) or a conventional CBL-only group (*n* = 60). The experimental group utilized reconstructed CT/MRI DICOM data rendered into interactive holographic models via Microsoft HoloLens 2 for real-time anatomical visualization and intraoperative guidance. Key outcome measures included post-training performance (theoretical and practical scores), self-directed learning ability, teaching satisfaction, critical thinking disposition, and knowledge retention at 1-, 3-, and 6-month follow-ups.

**Results:**

The combined teaching group demonstrated significantly superior outcomes across all measured domains (*P* < 0.05). Post-training scores were higher in theoretical knowledge (44.58 ± 2.09 vs. 40.28 ± 2.96), practical skills (44.63 ± 2.69 vs. 40.00 ± 2.64), and total score (89.21 ± 4.33 vs. 80.28 ± 5.55). Additionally, the group showed enhanced self-directed learning (4.38 ± 0.74 vs. 3.07 ± 0.73), higher teaching satisfaction (4.30 ± 0.74 vs. 3.15 ± 0.58), and improved critical thinking, notably in analyticity and cognitive maturity. Longitudinal assessments revealed better knowledge retention at all time points, with 6-month total scores of 76.92 ± 2.25 vs. 60.77 ± 5.97.

**Conclusions:**

Integrating 3D holographic reconstruction and intraoperative navigation into CBL teaching significantly improves urology residents’ comprehension, critical thinking, surgical preparedness, and long-term knowledge retention during laparoscopic partial nephrectomy training. This multimodal teaching model may serve as a valuable adjunct to traditional residency training frameworks.

## 1. Introduction

Standardized residency training plays a critical role in China’s healthcare reform, serving as a foundational mechanism for cultivating clinically competent physicians equipped with both strong theoretical knowledge and practical skills through structured clinical experience [1]. Traditional urology education has largely relied on two-dimensional imaging and static anatomical models—an approach that poses inherent limitations in developing spatial cognition. Unlike other surgical disciplines, urology presents distinct educational challenges due to its complex anatomical structures and the high level of precision required during surgical procedures [2]. The kidneys are located deep in the retroperitoneal space, with their anterosuperior surfaces adjacent to solid organs such as the liver, gallbladder, pancreas, and spleen, and their posterior aspects closely related to the psoas major and diaphragm muscles. Additionally, the vascular anatomy in this region is intricate, involving renal and adrenal veins and arteries and their proximity to the abdominal aorta and inferior vena cava. During residency training, it is commonly observed that residents struggle to comprehend the spatial relationships between renal tumors and adjacent anatomical structures-particularly in cases involving hilar and endophytic tumors-resulting in a steep learning curve. Facilitating a deeper understanding of renal tumor anatomy and improving residents’ proficiency in surgical techniques remains an urgent pedagogical challenge.

The advent of 3D digital holography and virtual reality systems has introduced new technological avenues for addressing these instructional limitations [3, 4]. Holographic imaging is a three-dimensional visualization technique based on optical principles that reconstructs two-dimensional medical images into virtual 3D models rich in anatomical detail. These models can be interacted with in real-time using mixed reality devices [5]. As an emerging technology in medical imaging, holography has revolutionized clinical diagnostics, surgical planning, and medical education [6, 7]. When combined with intraoperative imaging, this technology provides real-time guidance during surgery, improving both precision and safety [8, 9]. In the educational context, holographic imaging offers residents stereoscopic and highly intuitive anatomical views, significantly enhancing their understanding of complex anatomical structures—particularly in surgical skills training [10].

Case-based learning (CBL), a widely adopted teaching model in urology residency programs, emphasizes clinical scenarios to develop students’ diagnostic reasoning and problem-solving skills [11]. However, residents undergoing standardized training often face intense academic pressure and limited time for self-directed learning. Sole reliance on the CBL model may not sufficiently expose trainees to the breadth and complexity of clinical conditions. In surgical education, an overdependence on video-based resources can diminish students’ initiative and hinder the development of deep, independent thinking, ultimately impairing their critical thinking skills [12]. Moreover, such methods often lack effective guidance on actual surgical procedures.

Therefore, this study aims to evaluate the effectiveness and feasibility of integrating 3D digital holographic imaging with the CBL teaching model in the context of standardized urology residency training. By constructing detailed 3D holographic models of renal tumors, we seek to determine whether this approach can enhance residents’ anatomical understanding, surgical competence, and overall learning experience.

## Materials and methods

### Study design and participants

A total of 120 resident physicians who participated in the urology training at the Second People’s Hospital of Hefei City from August 2021 to May 2024 were selected and randomly divided into a combined teaching group and a conventional CBL-only group, all of whom passed the pre-training assessment. The combined teaching group consisted of 60 individuals, including 46 males and 14 females, aged 23–35 years, with an average age of (26.12 ± 3.14) years, and adopted the holographic imaging combined with CBL teaching model. The conventional CBL-only group consisted of 60 individuals, including 48 males and 12 females, aged 24–34 years, with an average age of (26.57 ± 2.59) years, and adopted the conventional CBL teaching model.

Inclusion criteria: (1) Possession of a medical bachelor’s degree or higher, with a clinical medicine professional degree and passing all assessment results. (2) Surgical residency trainees in their second and third years (3) Have not participated in standardized urology training. (4) Have not previously participated in any teaching related to holographic imaging technology. (5) Normal basic listening, speaking, reading, and writing abilities.

Exclusion criteria: (1) Residents who take leave or miss classes due to personal reasons during the training. (2) Have prior work or study experience related to the technology. (3) Are currently receiving or have previously received other related research. (4) Surgical residency trainees in their first year. (5) Other situations that researchers may consider to affect the experimental results. There were no statistically significant differences in baseline data between the two groups (*P* > 0.05), as shown in Table 1. Both groups were taught by the same senior instructor.

**Table 1:** Comparison of baseline characteristics and pre-course scores between the two groups

Group/characteristics	Combined teaching group (*n* = 60)	Individual teaching group (*n* = 60)	Statistical value	*P* value
Gender			0.20	0.658
Male	46 (76.7%)	48 (80.0%)		
Female	14 (23.3%)	12 (20.0%)		
Age (years)	26.12 ± 3.14	26.57 ± 2.59	− 0.86	0.393
Pre-course theoretical exam score	38.47 ± 2.66	38.73 ± 3.96	− 0.43	0.666
Pre-course practical skills score	37.90 ± 3.51	38.17 ± 4.69	− 0.35	0.725
Pre-course total score	76.37 ± 4.67	76.90 ± 5.55	− 0.57	0.570

### Teaching methodology

Both resident groups completed an identical ≥ 3-month standardized urology training curriculum supervised by the same senior faculty (attending/associate chief physician with ≥ 5 years’ surgical experience). The comprehensive program incorporated: (1) theoretical modules—including case-based discussions, renal anatomy review, and imaging interpretation; and (2) practical training—featuring simulated laparoscopic exercises, vascular dissection techniques, and suturing/renorrhaphy practice. This dual-component approach ensured uniform exposure to essential cognitive and psychomotor skill development while maintaining consistent instructional quality across groups.

#### Control group (CBL-only instruction)

The control group received a standardized case-based learning (CBL) curriculum without direct surgical involvement, delivered through: (1) didactic lectures utilizing 2D preoperative imaging (CT/MRI) to demonstrate renal tumor characteristics and laparoscopic partial nephrectomy principles; (2) structured case discussions covering diagnostic criteria, surgical indications/contraindications, and perioperative management; and (3) video-based surgical simulations emphasizing procedural workflow and critical operative steps—all strictly following urological surgery training guidelines while focusing on cognitive skill development rather than hands-on surgical performance.

#### Experimental group (integrated 3D holographic-CBL training)

**Fig. 1:**
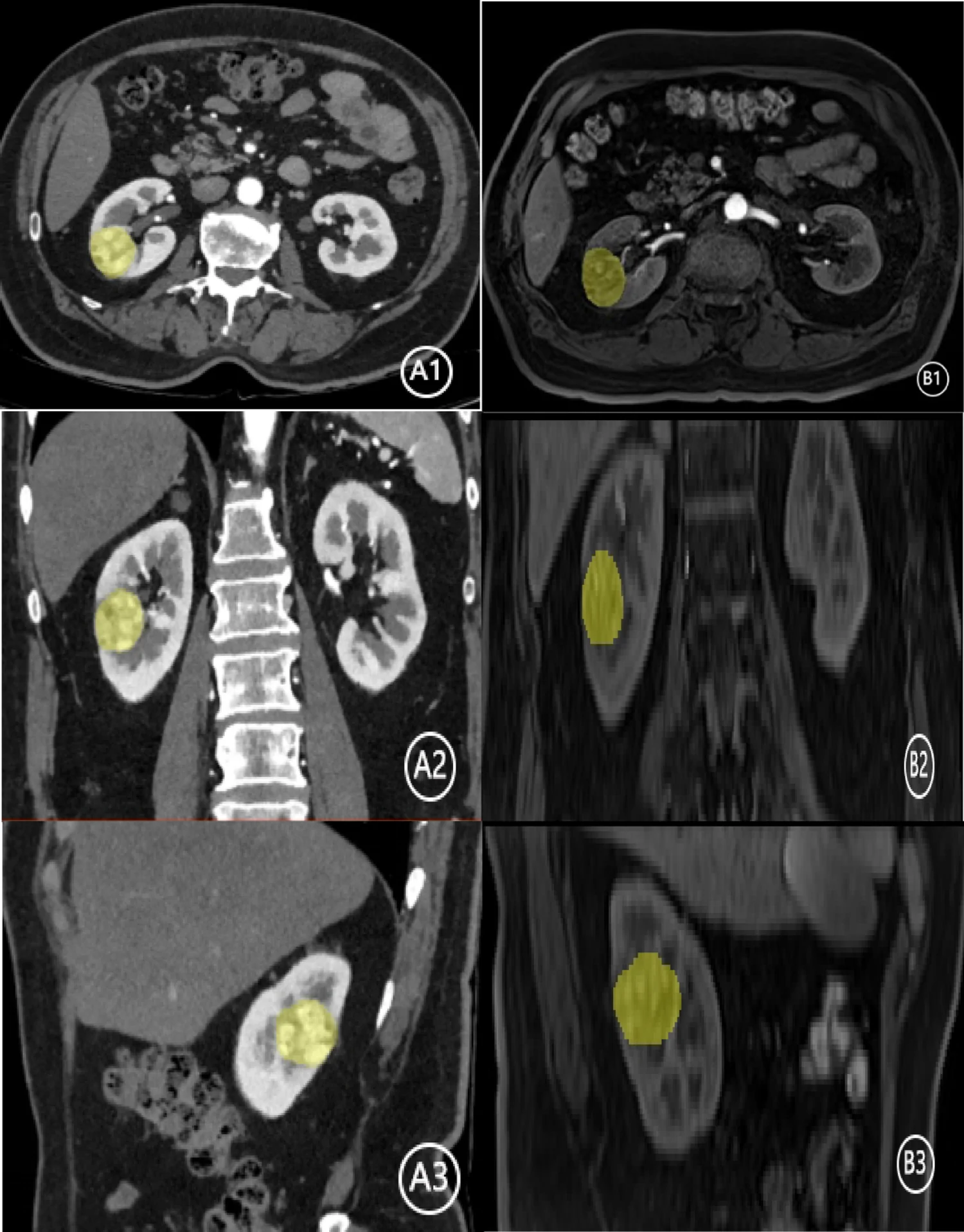
Original CT and MRI images (**A1**–**A3**: CT images; **B1**–**B3**: MRI images)

**Fig. 2:**
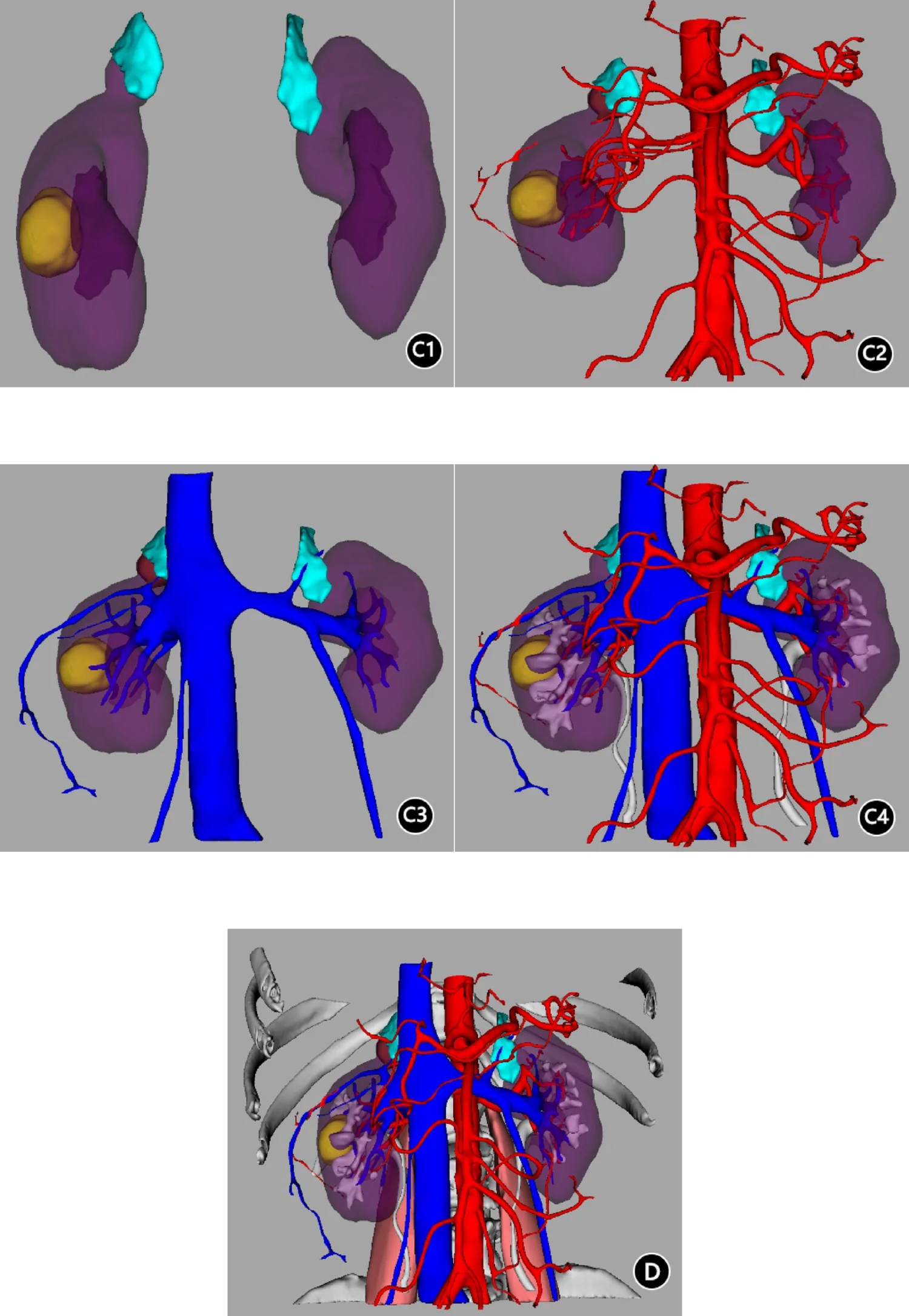
Reconstructed model demonstrating the relationship between the tumor and the kidney, blood vessels, and collecting system (**C1**: tumor lesion; **C2**: tumor-artery relationship; **C3**: tumor-vein relationship; **C4**: tumor-vasculature and collecting system relationship; **D**: complete holographic reconstruction model)

**Fig. 3:**
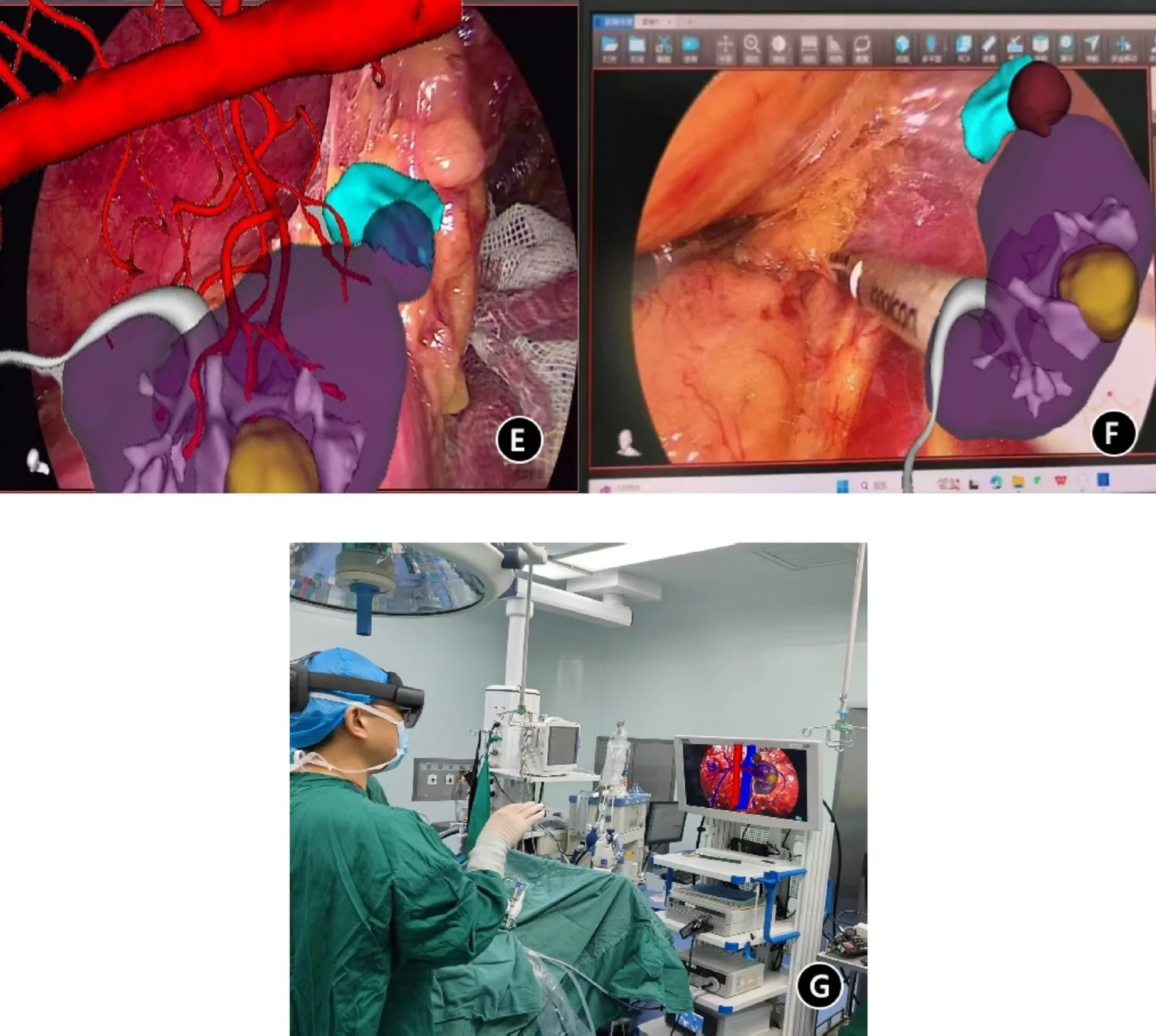
Holographic imaging for real-time navigation during laparoscopic partial nephrectomy (**E**–**F**: real-time navigation; **G**: mixed reality glasses guiding resident training)

The teaching content is supplemented with a holographic imaging system on the basis of the conventional CBL-only teaching group. The guiding physician acquires the DICOM data of the original CT and MRI of renal tumor patients (as shown in Fig. 1). After the data import is completed, image modeling is performed using computer automatic threshold segmentation and manual segmentation. The reconstructed DICOM format data was transmitted to the 3D post-reconstruction workstation (Extended Brilliance Workspace V4.5, Philips Medical Systems, Cleveland, Ohio), where two senior radiologists performed 3D kidney reconstruction using multi-planar reconstruction (MPR), volume rendering (VR), maximum intensity projection (MIP), and surface shaded display (SSD). Stereoscopic images of kidney anatomy and vasculature were obtained through the arbitrary plane cutting function. The edited models were then exported in a specific format via the mixed reality imaging system to acquire holographic imaging data for preoperative planning. By adjusting the model’s angle to display all relevant anatomical structures, including the kidney and its tumor lesions, renal arteries and veins, adrenal gland, and collecting system (as shown in Fig. 2). Finally, through mixed reality holographic imaging technology, the digitized information is holographically projected into the real physical environment, providing an intuitive and three-dimensional imaging experience. The attending physician utilizes the latest HoloLens 2 system and mixed reality glasses for real-time intraoperative navigation to identify tissues or organs that require focused attention during surgery, guiding resident physicians in learning and understanding laparoscopic partial nephrectomy, as illustrated in Fig. 3.

### Evaluation methods

#### Clinical teaching quality assessment

A research methodology combining literature review and the Delphi expert consultation method was employed. Through systematic literature review and two rounds of expert consultation, and with reference to the national urology residency standardized training assessment standards, a theoretical and practical skills assessment and evaluation system was constructed. The theoretical assessment module (total score: 50 points) consists of two parts: objective questions and case analysis questions. The objective questions section (40 points) includes: ① 20 basic theory single-choice questions (Type A1/A2); ② 10 clinical scenario single-choice questions (Type A3/A4); ③ 2 clinical cases with multiple-choice questions (indefinite number of choices), each case comprising 5 questions, totaling 10 items. The case analysis section includes one typical clinical case, focusing on the assessment of clinical thinking ability. The duration of the theoretical assessment is 60 min. The Skills Operation Assessment Module (total score: 50 points) focuses on the core clinical operation skills in urology, with an assessment duration of 30 min. All assessment questions are randomly selected from a standardized question bank that has been updated and validated by experts, ensuring the representativeness and scientific rigor of the assessment content. Additionally, a bar chart is drawn for comparison.

#### Evaluation of students’ self-directed learning ability and teaching satisfaction

This study was independently designed with reference to the medical self-directed learning ability assessment scale developed by Wang Xiaodan et al. [13], which has good reliability and validity (test-retest reliability = 0.951, Cronbach’s α = 0.907). A multi-dimensional evaluation framework was adopted, encompassing 2 first-level dimensions (subjective motivational beliefs, explicit behavioral performance), 6 s-level indicators (learning motivation, cognitive beliefs, goal planning ability, self-regulation ability, information processing ability, collaborative communication ability), and 30 specific assessment items. Scoring was conducted using the Likert 5-point scale (1 = "completely disagree” to 5 = "completely agree”), with higher total scores indicating stronger self-directed learning abilities among participants, as well as greater acceptance and sustained willingness to use the teaching model [14]. This scale comprehensively assesses the performance of resident physicians in self-directed learning under the new teaching model, and a bar chart is plotted for comparison.

#### Critical thinking assessment

After the training, a self-designed scale based on the California Critical Thinking Disposition Inventory was distributed to both groups of residents to quantitatively evaluate their critical thinking skills [15]. Higher scores indicate stronger traits in the respective items. This scale is capable of assessing the critical thinking of resident physicians and a bar chart was drawn for comparison.

#### Assessment of forgetting degree

At the end of the first, third, and sixth months after the training, theoretical and practical assessments are conducted for the resident physicians. The scores of the combined teaching group and the individual teaching group are compared to evaluate the forgetting rate of knowledge among the resident physicians. A line chart is drawn to reflect the knowledge forgetting rate.

## Results

### Comparison of assessment scores between groups

**Fig. 4:**
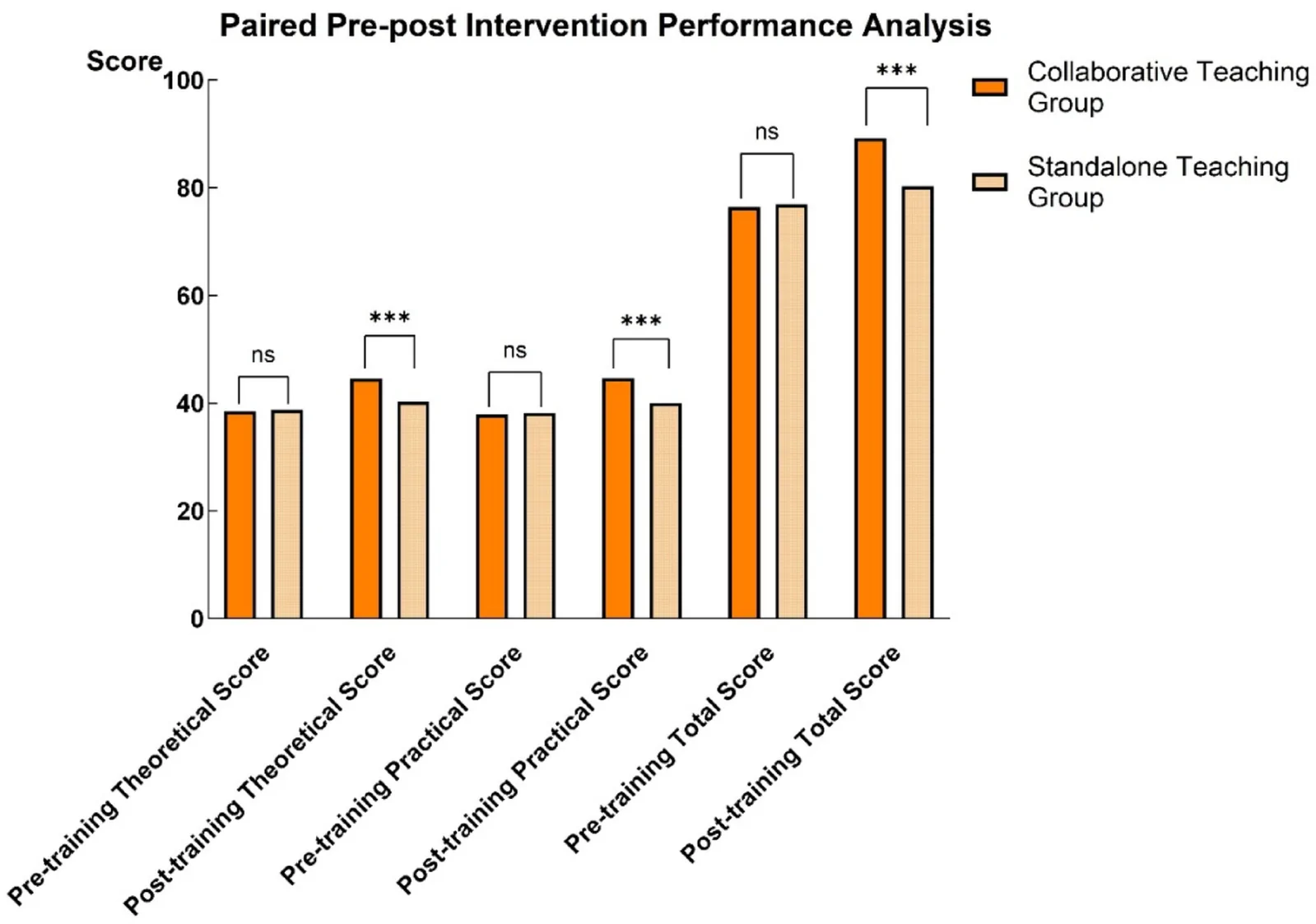
Paired pre-post intervention performance analysis (comparison of the theoretical scores before and after training, the practical scores before and after training, and the total scores before and after training between the two groups) (note: ***P< 0.001; ns *P* > 0.05).

As shown in Table 2; Fig. 4, combined teaching group achieved theoretical and practical scores of (44.58 ± 2.09) and (44.63 ± 2.69), respectively, with a total score of (89.21 ± 4.33). In contrast, the individual teaching group scored (40.28 ± 2.96) in theoretical knowledge and(40.00 ± 2.64) in practical skills, with a total score of (80.28 ± 5.55). The combined teaching group demonstrated significantly higher theoretical, practical, and total scores compared to the individual teaching group (*P* < 0.05).

**Table 2:** Comparison of post-training performance between the two groups

Assessment category	Combined teaching group (*n* = 60)	Individual teaching group (*n* = 60)	Statistical value	*P* value
Post-training theoretical score	44.58 ± 2.09	40.28 ± 2.96	9.19	< 0.001
Post-training practical score	44.63 ± 2.69	40.00 ± 2.64	9.53	< 0.001
Post-training total score	89.21 ± 4.33	80.28 ± 5.55	12.79	< 0.001

### Evaluation of self-directed learning ability and teaching satisfaction

**Fig. 5:**
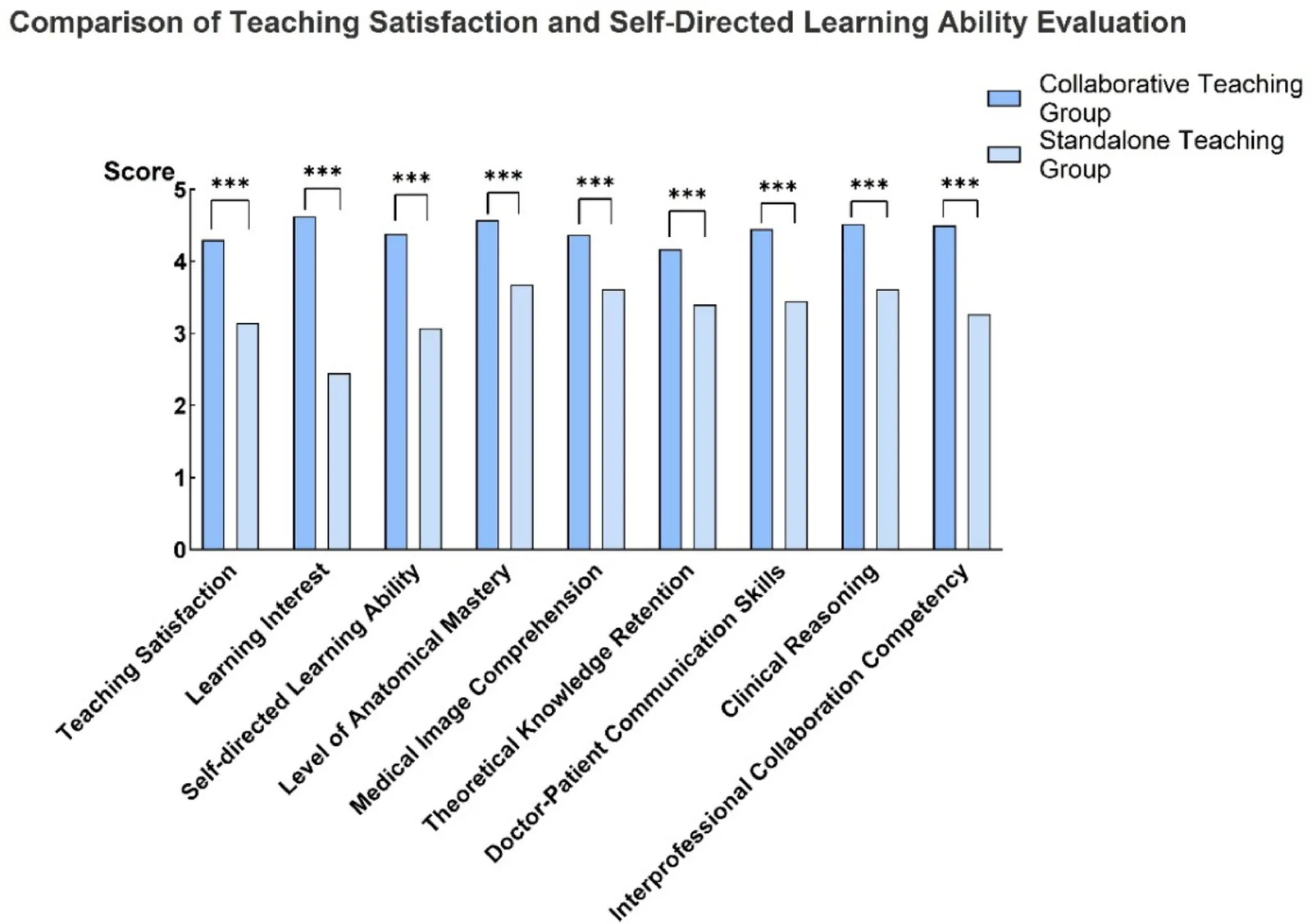
Comparison of teaching statisfaction and self-directed learning ability evaluation after training(including teaching satisfaciton, learning interest, self-directed learning ability, level of anatomical mastery, medical image comprehension, theoretical knowledge retention, doctor–patient communication, clinical reasoning ability, teamwork competency) (note: ***P<0.001; ns *P*>0.05)

As shown in Table 3; Fig. 5, The combined teaching group outperformed the control group in teaching satisfaction, learning interest, self-directed learning ability, anatomy proficiency, medical imaging comprehension, theoretical knowledge retention, doctor–patient communication, clinical reasoning ability, and teamwork ompetency, with statistically significant differences (*P* < 0.05).

**Table 3:** Comparison of post-training teaching satisfaction and self-directed learning competency between groups

Evaluation metrics	Combined teaching group (*n* = 60)	Individual teaching group (*n* = 60)	Statistical value	*P* value
Teaching satisfaction	4.30 ± 0.74	3.15 ± 0.58	9.47	< 0.001
Learning interest	4.63 ± 0.49	2.45 ± 0.96	15.66	< 0.001
Self-directed learning ability	4.38 ± 0.74	3.07 ± 0.73	9.80	< 0.001
Anatomy proficiency	4.57 ± 0.72	3.68 ± 0.65	7.04	< 0.001
Medical imaging comprehension	4.37 ± 0.74	3.62 ± 0.69	5.76	< 0.001
Theoretical knowledge retention	4.17 ± 0.67	3.40 ± 0.81	5.67	< 0.001
Doctor–patient communication	4.45 ± 0.70	3.45 ± 0.75	7.57	< 0.001
Clinical reasoning ability	4.52 ± 0.75	3.62 ± 0.85	6.18	< 0.001
Teamwork competency	4.50 ± 0.81	3.27 ± 0.84	8.17	< 0.001

### Critical thinking assessment

**Fig. 6:**
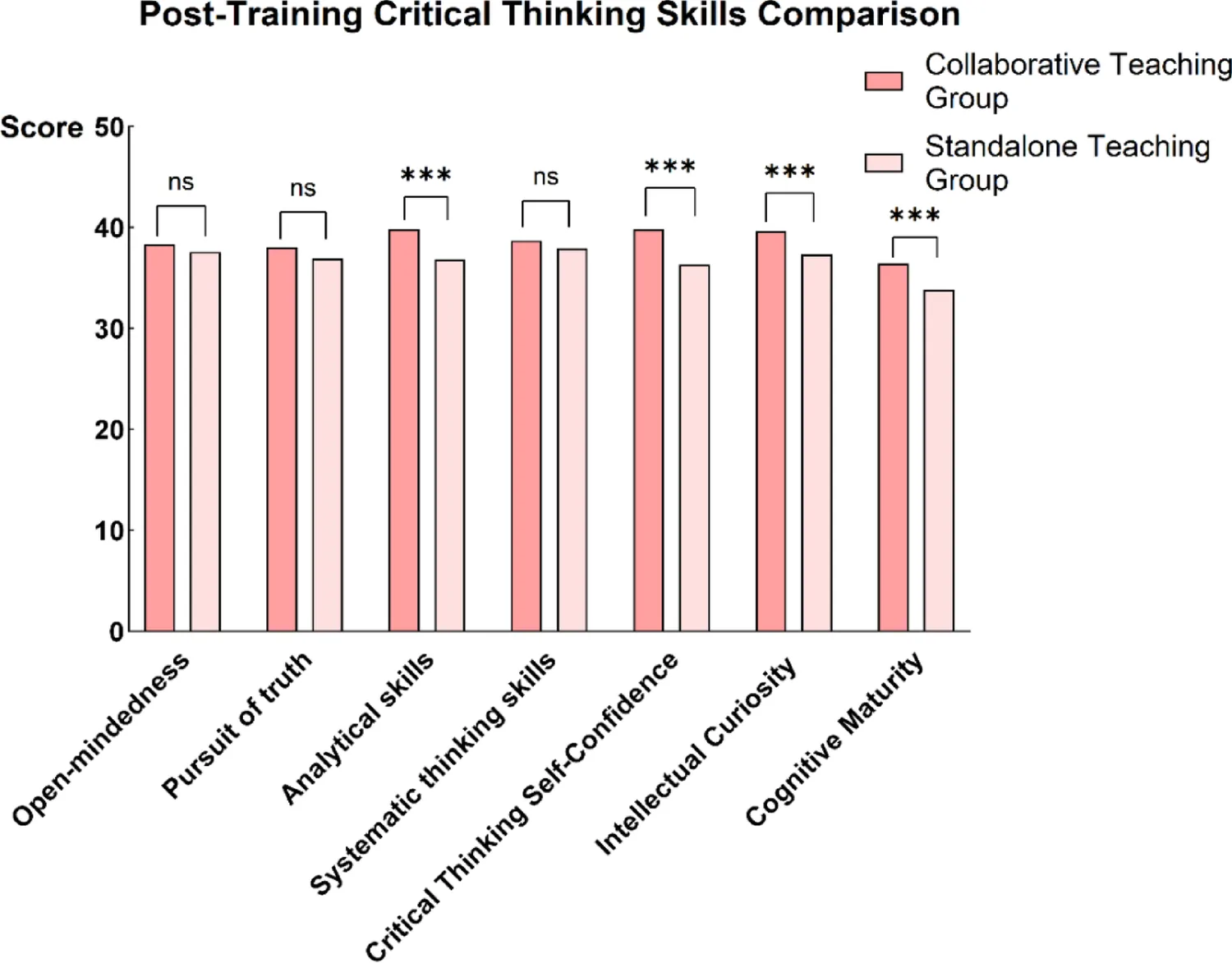
Post-training critical thinking skills comparison (including open-mindedness, truth-seeking, analyticity, systematicity, ct self-confidence, inquisitiveness and cognitive maturity) (note: ***P<0.01; ns *P* > 0.05)

As shown in Table 4; Fig. 6, The combined teaching group showed superior performance in analyticity, critical thinking self-confidence, inquisitiveness, and cognitive maturity compared to the individual teaching group (*P* < 0.05). While it also performed better in open-mindedness, truth-seeking, and systematicity, these differences were not statistically significant (*P* > 0.05).

**Table 4:** Comparison of critical thinking dispositions between teaching groups

Critical thinking dimension	Combined teaching group (*n* = 60)	Individual teaching group (*n* = 60)	Statistical value	*P* value
Open-mindedness	38.38 ± 3.04	37.60 ± 2.47	1.55	0.124
Truth-seeking	38.03 ± 3.35	36.93 ± 2.82	1.95	0.054
Analyticity	39.88 ± 2.41	36.85 ± 2.60	6.64	< 0.001
Systematicity	38.70 ± 2.36	37.93 ± 2.36	1.78	0.078
CT self-confidence inquisitiveness	39.87 ± 2.63	36.35 ± 2.22	7.93	< 0.001
Inquisitiveness	39.63 ± 2.11	37.37 ± 2.44	5.45	< 0.001
Cognitive maturity	36.43 ± 2.67	33.87 ± 1.91	6.06	< 0.001

### Knowledge retention assessment

**Fig. 7:**
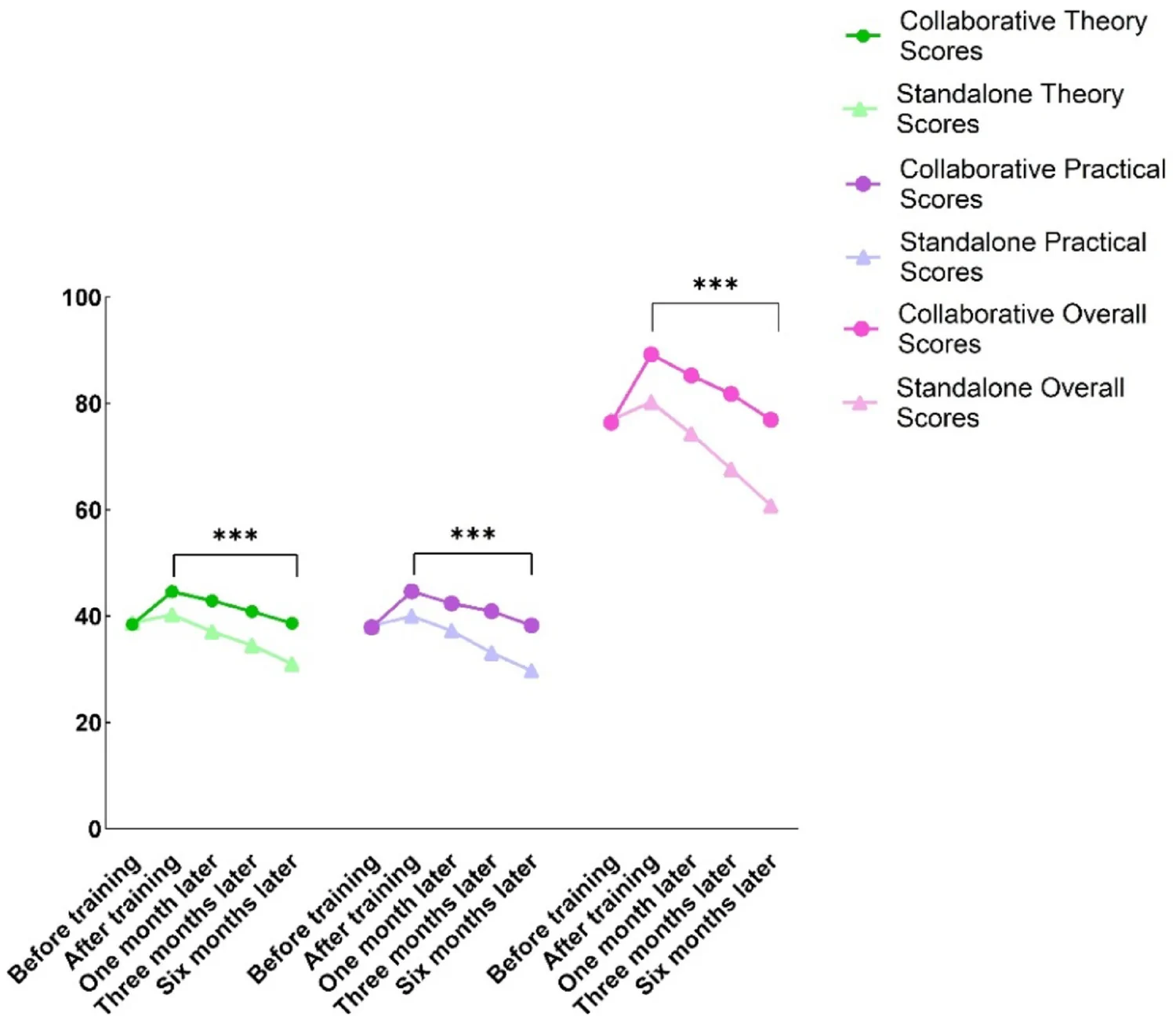
Line chart of score trends between groups (note: ***P < 0.001) (Comparison of scores before and after training, and comparisons of scores at 1 month, 3 months, and 6 months after training)

As shown in Table 5; Fig. 7,The combined teaching group demonstrated significantly better performance in theoretical knowledge, practical skills, and total scores at 1-, 3-, and 6-month follow-ups compared to the individual teaching group (all *P* < 0.05).

**Table 5:** Knowledge retention at 1-, 3-, and 6-month follow-ups after training completion

Assessment metrics	Combined teaching group (*n* = 60)	Individual teaching group (*n* = 60)	Statistical value	*P* value
1-month post-training				
Theoretical knowledge	42.88 ± 2.88	37.10 ± 1.73	13.32	< 0.001
Practical skills	42.38 ± 1.75	37.23 ± 2.53	12.98	< 0.001
Total score	85.27 ± 3.21	74.33 ± 3.00	19.29	< 0.001
3-month post-training				
Theoretical knowledge	40.85 ± 2.22	34.53 ± 4.78	9.29	< 0.001
Practical skills	40.90 ± 2.28	33.12 ± 3.45	14.59	< 0.001
Total score	81.75 ± 2.99	67.65 ± 5.81	16.71	< 0.001
6-month post-training				
Theoretical knowledge	38.65 ± 1.52	31.02 ± 4.51	12.43	< 0.001
Practical skills	38.27 ± 1.89	29.75 ± 3.03	18.49	< 0.001
Total score	76.92 ± 2.25	60.77 ± 5.97	19.62	< 0.001

## Discussion

Renal cancer is one of the common malignant tumors in the urinary system, and its incidence has been continuously increasing in recent years, ranking among the highest in the world [16]. Currently, laparoscopic partial nephrectomy (LPN) is the primary treatment for localized renal tumors and is considered the gold standard for treating localized renal cancer, widely applied in clinical practice [17].The procedure of laparoscopic partial nephrectomy is complex and technically challenging, especially for endophytic and hilar tumors. Conventional surgeons often rely solely on their experience to mentally convert two-dimensional images into three-dimensional representations preoperatively, which can easily lead to discrepancies with the intraoperative field of view and even result in missed lesions. During the teaching process, there exists the drawback of the surgical procedure not being intuitive. In the course of teaching, one can only verbally describe the techniques and key points of operation, making it difficult for students to form a mental image. Consequently, it is challenging to objectively understand the surgical process, making LPN surgery particularly difficult to master, often resulting in a long learning curve.

Standardized residency training, as a crucial phase in the clinical physician cultivation system, has a direct impact on the professional development trajectory of physicians [18]. In current clinical teaching practices, residents commonly face multiple pressures: on one hand, they need to complete heavy daily diagnostic and treatment tasks, and on the other hand, they must cope with various assessment evaluations. This high-intensity work and study state makes it difficult for them to systematically master the intricate structures of the urinary system through traditional anatomical atlases. Especially for surgeries such as LPN, due to the confined operative space and complex anatomical layers, relying solely on observing surgical videos makes it challenging for residents to accurately understand the key points of the procedure. Therefore, exploring innovative teaching methods and constructing efficient knowledge transfer models have become an urgent need to enhance the quality of urology specialty training.

In this context, the innovative application of holographic imaging technology has opened up new avenues for urology residency training. Compared to traditional surgical fields, urology has distinct professional characteristics, with its diagnostic and therapeutic processes predominantly guided by minimally invasive surgery and endoluminal treatment techniques, which impose higher demands on the professional competence of practitioners. Physicians not only need to master the intricate anatomical structures and vascular pathways of the urinary system but must also possess complex surgical operation skills and precise clinical decision-making abilities. This technology, through three-dimensional imaging, provides residents with an intuitive and interactive learning environment, enabling them to gain a deep understanding of complex anatomical relationships and simulate real surgical scenarios, thereby effectively enhancing their clinical operation skills and surgical techniques. This immersive training model has significantly improved the spatial cognitive limitations inherent in traditional teaching methods, providing crucial technical support for cultivating highly qualified professionals in urology.

The CBL teaching model, by being case-oriented, enhances the quality of teaching and strengthens the comprehensive abilities of trainees compared to traditional teaching models such as PBL [19, 20] However, compared to the conventional CBL-only teaching model, the holographic imaging combined with the CBL teaching model offers a new approach. The limited anatomical resources in most hospitals constrain the in-depth understanding and mastery of anatomical knowledge by resident physicians. Although conventional CBL-only teaching model is superior to traditional teaching models, being solely case-oriented, it makes it difficult for resident physicians to understand and fails to stimulate their interest in learning [21]. For beginners, the anatomical location of renal tumors and their surrounding vessels is complex and difficult to comprehend, and the surgical procedure of laparoscopic partial nephrectomy is intricate and challenging. The integration of holographic imaging technology with the CBL teaching model, through specific surgical cases combined with stereoscopic and intuitive three-dimensional images, allows instructors to use holographic imaging technology to present the patient’s kidney and its surrounding structures in three dimensions during the teaching process. This enables more accurate surgical path planning and avoids damage to important vessels and nerves [22, 23]. It can help resident physicians more intuitively understand complex anatomical structures and surgical pathways, thereby stimulating their learning interest [24]. Secondly, the application of holographic imaging technology in preoperative planning and intraoperative navigation can enhance the precision and safety of surgeries [25, 26].Through holographic imaging technology, the vascular distribution and course in the renal region, as well as the spatial configuration of the urinary collecting system and adjacent tissues, can be realistically reproduced.

In the virtual training for laparoscopic partial nephrectomy (LPN), this technology not only allows resident physicians to repeatedly practice procedures under zero-risk conditions but also dynamically presents the anatomical hierarchical relationships of the surgical field through a multi-dimensional visualization interface. This immersive training mode offers dual advantages: on one hand, it enables trainees to establish accurate spatial cognition and master key operational points before actual surgery; on the other hand, its real-time navigation function provides anatomical positioning references during actual surgery, effectively avoiding the risk of damage to important tissue structures. The intraoperative holographic navigation atlas generated by the system complements the laparoscopic view, helping surgeons quickly establish three-dimensional correspondences of anatomical landmarks. This visual aid significantly shortens the learning curve and has a positive impact on improving surgical precision and patient safety. Holographic imaging can also be applied in teaching for doctor–patient communication, by visually presenting the anatomical location, volume parameters of lesions, and their spatial relationship with adjacent tissues, enabling patients and their families to have a more accurate understanding of the condition. This visual communication model not only significantly enhances patients’ acceptance of treatment plans but also provides an innovative practical platform for standardized doctor–patient communication teaching. In this study, the combined teaching group outperformed the separate teaching group in terms of assessment scores, self-directed learning ability, teaching satisfaction, critical thinking, and the degree of forgetting, to a certain extent indicating the effectiveness of this model and its acceptance and recognition by urology resident physicians. Although limited by single-center research and a small sample size, it still holds potential prospects.

The notable advantages observed in the joint teaching group can likely be attributed to the unique cognitive and instructional benefits of 3D holographic visualization technology. Firstly, it directly enhances spatial cognition by providing intuitive, rotatable 3D models of kidney anatomy, which alleviates the significant cognitive load associated with reconstructing complex structures from 2D CT/MRI images in the mind. This immersive multisensory experience aligns with Kolb’s experiential learning theory, placing residents in concrete learning experiences and promoting deeper information processing and more robust memory formation [27]. Additionally, the technology effectively bridges the gap between theoretical knowledge and clinical practice. By enabling residents to interact with patient-specific anatomical models during case discussions, it establishes a direct visual link between “what” (anatomy and pathology) and “how” (surgical approaches and techniques), thereby facilitating the transfer of learning outcomes to surgical scenarios.

Our findings align with a growing body of literature demonstrating the benefits of VR and AR simulators in surgical education, particularly in enhancing procedural knowledge and psychomotor skills [28–31]. However, the mixed reality platform employed in this study offers unique pedagogical advantages. Unlike fully immersive VR systems that isolate users entirely within a virtual environment, our HoloLens-based MR approach seamlessly superimposes digital holograms onto the real physical world. This preserves crucial haptic feedback and enables uninterrupted interaction with instructors and peers, fostering a collaborative learning environment that more closely mirrors the essence of teamwork in an actual operating room. This critical difference may render MR a more suitable platform for integrating cognitive and psychomotor training, as it can support the cultivation of situational awareness and team collaboration skills while facilitating the acquisition of technical skills.

China’s standardized residency training program was modeled after the American system, sharing some similarities in curriculum design. As long as the surgical learning gradient curve is followed and corresponding courses are set up to train surgical residents, there is potential for widespread adoption, as the principles of holographic imaging and the CBL educational model are applicable to any country. Education for surgeons at different stages can be conducted through tiered and gradient approaches, with continuous optimization of the educational process and details through the implementation of satisfaction questionnaires. Additionally, a dedicated residency training camp can be established to train holographic interactive sensory experiences, aiming to find and simulate the sensation of surgery. Previous studies have demonstrated that holographic imaging technology can be applied in the education of urology, orthopedics, gynecology, and surgical nursing [32–38]. This is precisely because complex three-dimensional anatomy requires significant surgical precision, and embracing such technology can position one at the forefront of development. Subsequent promotion can categorize learners of different qualifications into various gradients, such as junior residents and those who have undergone systematic training, for further research. Crucially, we now emphasize the unique advantages of our mixed reality approach using HoloLens 2. The advantages of applying holographic imaging in teaching include: It creates immersive training environments that replicate professional settings, allowing learners to visualize abstract concepts and simulate the spatial and tactile elements of real tasks. Its real-time interactivity provides immediate feedback, empowering learners to self-direct their progress without the need for constant instructor supervision. Unlike fully immersive VR and AR, which isolates users completely, MR allows residents to interact simultaneously with digital holograms and the physical environment (e.g., simulators, instructors, peers). This preserves the crucial tactile feedback and team-based learning, potentially offering a more seamless and effective pathway for transitioning to the actual operating room compared to fully virtual simulations.

This study still has some limitations. Although this study was conducted within a single institution in China, the educational principles and technological tools employed—3D holographic reconstruction and CBL—are broadly applicable across diverse residency training systems. The challenges of mastering complex surgical anatomy and procedural skills are not unique to any one country, and the immersive, interactive nature of holographic visualization can enhance spatial cognition and operative preparedness irrespective of geographic setting. However, differences in training duration, case volume, institutional resources, and curricular structure may affect the extent of its implementation and effectiveness. Future multi-center international studies are warranted to validate these findings across different educational environments. Additionally, we must acknowledge the potential existence of a novelty bias. The enthusiasm generated by the use of cutting-edge technologies such as HoloLens 2 may have temporarily heightened the motivation and engagement levels of the experimental group, potentially exaggerating the short-term outcomes. Although this is a common challenge when evaluating innovative teaching tools, future long-term studies are crucial to determine whether these benefits can be sustained after the technology becomes routine(the novelty of the technology wears off). The cost of the holographic imaging system is high, and its operation is complex. The processing and analysis of data require professional technical personnel and extensive clinical experience, making it difficult to widely promote and apply. This research is a single-center study with a limited sample size and is only targeted at resident physicians. Future studies with larger scales are needed to enhance the reliability of the conclusions, and more educational practices are required to explore the universality of this model.

The evidence from this study provides clear implications for the curriculum design of urology residency training. The 3D holographic-CBL model can be strategically integrated as a high-fidelity simulation module, serving as a mandatory component for preoperative planning of complex partial nephrectomy. It can also be utilized as a “surgical rehearsal” tool for residents before they assist in or perform specific procedures, thereby reducing the learning curve and enhancing patient safety. Looking ahead, the following future research directions are of paramount importance. First, multicenter, international trials are needed to validate the effectiveness of this model across different educational systems. Second, long-term follow-up studies are crucial to assess skill retention and its translation into real-world surgical performance and patient outcomes. Finally, conducting research on the cost-effectiveness of this technology and determining the optimal “dosage” and timing of holographic training within comprehensive surgical curricula are crucial for its rational implementation.

## Conclusion

In conclusion, the integration of 3D digital holographic imaging reconstruction and intraoperative navigation technology with the CBL teaching model can, to a certain extent, enhance the understanding of complex renal tumor structures among resident physicians. This approach is beneficial for improving the teaching effectiveness of LPN, fostering students’ autonomous learning and critical thinking skills, and aiding in the reduction of knowledge attrition. It represents a relatively ideal teaching methodology.

## References

[1] Cui Y, Wang T (2016) From the residency training in the United States to see the challenges and directions of China residency standardized training. Zhongguo Fei Ai Za Zhi 19(6):321–327 10.3779/j.issn.1009-3419.2016.06.03 PMC6015204 27335288

[2] Fisch R, Kuchakulla M, Tzeng M et al (2025) Important concepts in building a positive culture in a residency program. Curr Urol Rep 26(1):48 10.1007/s11934-025-01273-5 40459811

[3] d’Aiello AF, Cabitza F, Natali C et al (2023) The effect of holographic heart models and mixed reality for anatomy learning in congenital heart disease: an exploratory study. J Med Syst 47(1):64 10.1007/s10916-023-01959-8 PMC10191923 37195484

[4] D’Aiello AF, Bognoni L, Bevilacqua F et al (2023) Holographic techniques as a novel method for intervention planning: a tertiary centres experience. Curr Health Sci J 49(4):584–593 10.12865/CHSJ.49.04.15 PMC10976210 38559839

[5] Qi Z, Li Y, Xu X et al (2021) Holographic mixed-reality neuronavigation with a head-mounted device: technical feasibility and clinical application. Neurosurg Focus 51(2):E22 10.3171/2021.5.FOCUS21175 34333462

[6] Fujiki T, Nishimura R, Mase S et al (2019) Accurate detection of renal leukemic involvement in children using 3-D computed tomography modeling. Pediatr Int 61(7):679–687 10.1111/ped.13907 31173414

[7] Elhadidi M, Aldahouk A, Shawky M, Elbehairy MS, Atef M, El-Gengehi M (2019) Computer-guided calvarial mono-cortical bone blocks harvest: A novel approach for three-dimensional alveolar reconstruction of atrophic maxilla. Clin Implant Dent Relat Res 21(1):85–93 10.1111/cid.12714 30618184

[8] Liu C, Dong Z, Sheng M, Huang X, Huang Y (2024) Effects of PCNL under the guidance of hologram technology on stress response and renal injury factors in patients with complex kidney stones. J Med Biochem 43(4):469–479 10.5937/jomb0-48327 PMC11318061 39139180

[9] Zhang K, Wang L, Sun Y et al (2022) Combination of holographic imaging with robotic partial nephrectomy for renal hilar tumor treatment. Int Urol Nephrol 54(8):1837–1844 10.1007/s11255-022-03228-y 35568753

[10] Robinson BL, Mitchell TR, Brenseke BM (2020) Evaluating the use of mixed reality to teach gross and microscopic respiratory anatomy. Med Sci Educ 30(4):1745–1748 10.1007/s40670-020-01064-2 PMC7433990 32837799

[11] Qamar K, Rehman S, Khan MA (2016) Effectiveness of Case-Based learning during small groups sessions at army medical college. J Coll Physicians Surg Pak 26(3):232–233 26975961

[12] Basu Roy R, McMahon GT (2012) Video-based cases disrupt deep critical thinking in problem-based learning. Med Educ 46(4):426–435 10.1111/j.1365-2923.2011.04197.x 22429179

[13] Ladhania R, Haviland AM, Venkat A, Telang R, Pines JM (2021) The effect of medicaid expansion on the nature of new enrollees’ emergency department use. Med Care Res Rev 78(1):24–35 10.1177/1077558719848270 31132912

[14] Prachanukool T, Atiksawedparit P, Senasu S et al (2025) Comparing approaches to code status conversations between Thai and American emergency clinicians: a survey study. Emerg Med J 10.1136/emermed-2024-213883 PMC12513196 40518175

[15] Geyh S, Müller R, Peter C et al (2011) Capturing the psychologic-personal perspective in spinal cord injury. Am J Phys Med Rehabil 90(11 Suppl 2):S79–96 10.1097/PHM.0b013e318230fb68 21975679

[16] Young M, Jackson-Spence F, Beltran L et al (2024) Renal cell carcinoma. Lancet 404(10451):476–491 10.1016/S0140-6736(24)00917-6 39033764

[17] Bahadoram S, Davoodi M, Hassanzadeh S, Bahadoram M, Barahman M, Mafakher L (2022) Renal cell carcinoma: an overview of the epidemiology, diagnosis, and treatment. G Ital Nefrol 39(3):2022 vol3 [pii] 35819037

[18] Olde Bekkink M, Farrell SE, Takayesu JK (2018) Interprofessional communication in the emergency department: residents’ perceptions and implications for medical education. Int J Med Educ 9:262–270 10.5116/ijme.5bb5.c111 PMC6387781 30368487

[19] Lutsky K, Glickel SZ, Weiland A, Boyer MI (2013) What every resident should know about wrist fractures: case-based learning. Instr Course Lect 62:181–197 23395024

[20] Thistlethwaite JE, Davies D, Ekeocha S et al (2012) The effectiveness of case-based learning in health professional education. A BEME systematic review: BEME guide 23. Med Teach 34(6):e421–e444 10.3109/0142159X.2012.680939 22578051

[21] Woodham LA, Ellaway RH, Round J, Vaughan S, Poulton T, Zary N (2015) Medical student and tutor perceptions of video versus text in an interactive online virtual patient for problem-based learning: a pilot study. J Med Internet Res 17(6):e151 10.2196/jmir.3922 PMC4526950 26088435

[22] Cao Z, Xiu Y, Yu D, Li X, Yang C, Li Z (2023) Clinical value of mixed Reality-Assisted puncture navigation for percutaneous nephrolithotripsy. Urology 176:219–225 10.1016/j.urology.2022.12.067 36921844

[23] Wang L, Zhao Z, Wang G et al (2022) Application of a three-dimensional visualization model in intraoperative guidance of percutaneous nephrolithotomy. Int J Urol 29(8):838–844 10.1111/iju.14907 35545290

[24] Peng Y, Xie Z, Chen S et al (2023) Application effect of head-mounted mixed reality device combined with 3D printing model in neurosurgery ventricular and hematoma puncture training. BMC Med Educ 23(1):670 10.1186/s12909-023-04659-6 PMC10506205 37723452

[25] Antonelli A, Veccia A, Palumbo C et al (2019) Holographic reconstructions for preoperative planning before partial nephrectomy: a head-to-head comparison with standard CT scan. Urol Int 102(2):212–217 10.1159/000495618 30540991

[26] Shi X, Yang B, Guo F et al (2025) Artificial intelligence based augmented reality navigation in minimally invasive partial nephrectomy. Urology 199:20–26 10.1016/j.urology.2025.02.033 40010571

[27] Hung LY, Wang SM, Yeh TK (2023) Kolb’s experiential learning theory and marine debris education: effects of different stages on learning. Mar Pollut Bull 191:114933 10.1016/j.marpolbul.2023.114933 37119586

[28] Plotzky C, Lindwedel U, Sorber M et al (2021) Virtual reality simulations in nurse education: A systematic mapping review. Nurse Educ Today 101:104868 10.1016/j.nedt.2021.104868 33798987

[29] Foronda CL, Fernandez-Burgos M, Nadeau C, Kelley CN, Henry MN (2020) Virtual simulation in nursing education: a systematic review spanning 1996 to 2018. Simul Healthc 15(1):46–54 10.1097/SIH.0000000000000411 32028447

[30] Cao C, Cerfolio RJ (2019) Virtual or augmented reality to enhance surgical education and surgical planning. Thorac Surg Clin 29(3):329–337 10.1016/j.thorsurg.2019.03.010 31235302

[31] Hirt J, Beer T (2020) Use and impact of virtual reality simulation in dementia care education: a scoping review. Nurse Educ Today 84:104207 10.1016/j.nedt.2019.104207 31669968

[32] Siff LN, Mehta N (2018) An interactive holographic curriculum for urogynecologic surgery. Obstet Gynecol 132(Suppl 1):27S–32S 10.1097/AOG.0000000000002860 30247304

[33] Hall AJ, Walmsley P (2023) Technology-enhanced learning in orthopaedics: virtual reality and multi-modality educational workshops may be effective in the training of surgeons and operating department staff. Surgeon 21(4):217–224 10.1016/j.surge.2022.04.009 35624020

[34] Möller H, Creutzfeldt J, Valeskog K et al (2022) Technology-enhanced learning of human trauma biomechanics in an interprofessional student context. Teach Learn Med 34(2):135–144 10.1080/10401334.2021.1893735 33792438

[35] Hu X, Wang Y, Li J et al (2023) Three-Dimensional multimodality image reconstruction as teaching tool for Case-based learning among medical postgraduates: a focus on primary pelvic bone tumour education. BMC Med Educ 23(1):944 10.1186/s12909-023-04916-8 PMC10717389 38087270

[36] Tepper OM, Rudy HL, Lefkowitz A et al (2017) Mixed reality with hololens: where virtual reality Meets augmented reality in the operating room. Plast Reconstr Surg 140(5):1066–1070 10.1097/PRS.0000000000003802 29068946

[37] Bouvette MJ, Lee B, Bradley N (2025) Robotic simulation in urology training: implementation, curricula, and barriers across U.S. Residency programs. J Robot Surg 19(1):406 10.1007/s11701-025-02591-z 40684419

[38] Bodur G, Turhan Z, Altun YE et al (2025) Evaluating the effectiveness of virtual reality simulation in Cpr training for nursing students: a randomized controlled trial. Nurse Educ Pract 87:104486 10.1016/j.nepr.2025.104486 40743700

